# Tea Bioactive Compounds in Obesity Prevention and Management: Processing-Dependent Composition, Molecular Mechanisms, Human Evidence, and Translational Challenges

**DOI:** 10.3390/ijms27167203

**Published:** 2026-08-12

**Authors:** Yangxian Hu, Guoyuan Huang, Kwon Soonjae

**Affiliations:** 1 College of Physical Education, Sichuan Agricultural University, Ya’an 625014, China; 202207336@stu.sicau.edu.cn (Y.H.);; 2 Ya’an Key Laboratory of Sports Human Science and National Physical Fitness Promotion, Ya’an 625014, China; 3 Department of Taekwondo, Honam University, Gwangju 62399, Republic of Korea

**Keywords:** tea, obesity, catechins, clinical trials, bioavailability, gut microbiota, energy metabolism

## Abstract

Obesity is a heterogeneous chronic disease for which safe, scalable adjuncts to lifestyle and clinical care remain needed. Tea derived from *Camellia sinensis* contains catechins, caffeine, theaflavins, thearubigins, theabrownins, polysaccharides, and other constituents whose abundance is shaped by withering, fixation, partial oxidation, full oxidation, and post-fermentation. This review integrates processing-dependent composition with molecular mechanisms, gut–liver signaling, human evidence, safety, and real-world preparation. Evidence is strongest for modest effects of green-tea catechin–caffeine preparations on energy metabolism and selected anthropometric or lipid outcomes, whereas inhibition of adipogenesis, activation of AMP-activated protein kinase, browning of white adipose tissue, and many appetite-related pathways remain supported mainly by cell and rodent studies. A recent meta-analysis in women with overweight or obesity estimated mean reductions of −1.23 kg in body weight and −3.46 cm in waist circumference, with intervention durations across the included trials typically ranging from 4 to 24 weeks, though most of the evidence derives from short- to medium-term interventions (generally ≤12 weeks); heterogeneity was moderate to high and the average weight effect remained below conventional clinical thresholds. Partially oxidized oolong tea and fully oxidized or post-fermented teas provide distinct profiles of caffeine, oxidized polyphenols, and microbial metabolites; their metabolic effects are promising but are less consistently tested in adequately powered human trials. Across tea types, convergent mechanisms include reduced digestive-enzyme activity, increased fatty-acid oxidation, modulation of thermogenesis, reinforcement of the intestinal barrier, and microbiota-dependent production of short-chain fatty acids and bile-acid signals. Translation is constrained by low systemic polyphenol exposure (i.e., limited bioavailability of intact catechins and their metabolites in circulation due to poor intestinal absorption, extensive phase-II metabolism, and rapid clearance), non-standardized doses and products, short intervention periods, interindividual variability, and limited direct comparisons among tea types. Available clinical data do not support tea as a primary treatment for obesity; rather, unsweetened tea or standardized preparations may serve as adjuncts to evidence-based dietary, physical activity, behavioral, and medical management. Priority areas include physiologically relevant dosing, standardized reporting of brewed-tea composition, long-term trials, and microbiome-informed personalization.

## 1. Introduction

Obesity results from sustained perturbations in energy intake, substrate partitioning, adipose-tissue biology, neuroendocrine signaling, and environmental exposures rather than from a single behavioral cause. Expansion and dysfunction of white adipose tissue promote insulin resistance, ectopic lipid deposition, chronic low-grade inflammation, and cardiometabolic complications. Contemporary models therefore complement the conventional energy-balance framework with altered fuel partitioning, adipocyte differentiation, and endocrine-disrupting exposures [1,2,3,4]. Effective management usually requires coordinated dietary, physical activity, behavioral, pharmacological, or surgical approaches, while foods and beverages with reproducible metabolic effects may provide supportive—rather than substitutive—benefits.

Tea produced from *Camellia sinensis* is a globally consumed beverage that contains polyphenols, purine alkaloids, amino acids, polysaccharides, pigments, and volatile compounds. Previous reviews, including the comprehensive synthesis by Xu et al., established that tea can influence lipid absorption, energy expenditure, adipogenesis, inflammation, and the gut microbiota [5,6]. However, three gaps remain important for translation. First, tea is often treated as a uniform exposure even though processing converts catechins into chemically and biologically distinct oxidized or microbially transformed products. Second, mechanistic findings from cells and rodents are frequently discussed alongside human outcomes without distinguishing the evidence level or physiologically achievable exposure. Third, the composition of the beverage actually consumed depends on brewing temperature, time, leaf-to-water ratio, repeated infusion, and additions such as milk or sugar.

Accordingly, this review differs from earlier syntheses by linking processing-dependent chemistry to bioavailability, cellular targets, microbiota and bile-acid signaling, human efficacy, safety thresholds, and real-world preparation. The scope is restricted to true tea (*Camellia sinensis*); studies of unrelated herbal infusions are excluded. White tea is included as a minimally processed, non-oxidized reference; green tea as a non-oxidized, heat-fixed tea; oolong tea as a partially oxidized tea; black tea as a fully oxidized tea; and dark tea as a post-fermented product shaped by microbial transformation [7]. This review also evaluates whether statistically detectable changes in body weight are clinically meaningful and identifies priorities for clinicians, researchers, product developers, and consumers.

## 2. Processing-Dependent Composition, Chemical Characteristics, and Bioavailability

### 2.1. Catechins in White and Green Tea

Catechins are flavan-3-ols that occur predominantly as (–)-epigallocatechin-3-gallate (EGCG), epigallocatechin, epicatechin gallate, and epicatechin. White tea undergoes limited handling and drying, whereas green tea is rapidly heat-fixed to inactivate polyphenol oxidase; both therefore retain comparatively high concentrations of native catechins. Galloylation and hydroxylation influence hydrogen bonding, enzyme interactions, redox behavior, and membrane permeability, which helps explain the comparatively high activity of EGCG in experimental systems [8]. Nevertheless, compositional values vary widely by cultivar, leaf age, agronomy, storage, and preparation, making fixed percentages inappropriate unless measured in the specific product under study.

Oral exposure to intact catechins is limited by chemical instability, intestinal permeability, efflux transport, phase-II metabolism, hepatic first-pass clearance, and microbial biotransformation. Obesity may further reduce systemic exposure; in a pharmacokinetic comparison, obese participants exhibited lower plasma catechin exposure after a standardized green-tea confection than non-obese participants [9]. Consequently, mechanisms observed at tens of micromoles per liter in cell culture cannot be assumed to occur after customary tea drinking, where circulating intact EGCG is generally in the low-micromolar or submicromolar range. Conjugated metabolites, microbial ring-fission products, and local intestinal actions must therefore be considered alongside the parent compound [10].

### 2.2. Caffeine and Other Purine Alkaloids

Caffeine is present in all major tea classes, although its concentration is influenced more by genotype, leaf maturity, and extraction than by oxidation alone. By antagonizing adenosine receptors, caffeine can increase sympathetic activity, catecholamine signaling, lipolysis, and acute thermogenesis. Theobromine and theophylline contribute related but generally weaker effects [11]. Caffeine also interacts with catechins: catechins may prolong catecholamine action through catechol-O-methyltransferase-related pathways and modify digestive or microbial processes, whereas caffeine increases neural and peripheral lipolytic signaling. In animal models, low-dose EGCG plus caffeine has shown greater metabolic activity than either component alone and altered microbial and bile-acid profiles [12]. Human responses, however, depend on habitual caffeine intake, tolerance, sleep, genetics, and the comparator beverage.

### 2.3. Theaflavins, Thearubigins, and Theabrownins

During oolong and black tea manufacture, controlled enzymatic oxidation converts catechins through quinone intermediates into theaflavins and higher-molecular-weight thearubigins; post-fermentation of dark tea introduces additional microbial reactions and generates complex pigments, including theabrownins. These transformations are continuous rather than binary and are shaped by oxygen exposure, rolling, moisture, temperature, and fermentation microbiota [13,14,15,16]. Theaflavins can inhibit pancreatic lipase and regulate hepatic lipid metabolism in experimental models, whereas larger polymers may act primarily in the intestinal lumen or after microbial degradation [17].

The systemic bioavailability of theaflavins is low, partly because of poor epithelial permeability and transporter-mediated efflux [18]. This limitation does not imply inactivity: retained compounds can interact with digestive enzymes, bile acids, intestinal epithelial cells, and the microbiota. Black-tea polymers are chemically heterogeneous, and their microbial depolymerization may yield smaller phenolic metabolites with different absorption and signaling properties [19]. For dark tea, microbial fermentation also produces tea-specific metabolites whose identity and abundance depend on the manufacturing ecosystem; hence, conclusions should not be generalized across all post-fermented products.

### 2.4. White Tea as a Minimally Processed Reference and Comparison Across Tea Types

White tea provides a useful non-oxidized reference because it retains native catechins while undergoing less heat fixation than green tea. Comparative animal work suggests that white and dark tea can both attenuate diet-induced metabolic dysfunction but may do so through different compositional and microbiome signatures [20]. A recent human study reported improvements in anthropometric and metabolic variables after white-tea consumption, but replication with blinded, compositionally standardized interventions is required [21]. In cultured human subcutaneous preadipocytes and adipocytes, a white-tea extract reduced triglyceride incorporation, increased glycerol release, and downregulated peroxisome proliferator-activated receptor γ (PPARγ), CCAAT/enhancer-binding protein α (C/EBPα), and sterol regulatory element-binding protein 1c (SREBP-1c), indicating inhibition of adipogenesis and stimulation of lipolysis [22]. However, the experiment used a concentrated extract in vitro, including 50 μM EGCG in one component analysis, so it does not establish that customary white-tea consumption produces equivalent adipose-tissue exposure or clinical weight loss. Thus, white tea should not be assumed to be superior to green tea; its principal value in the present framework is to clarify how minimal processing, oxidation, and post-fermentation alter exposure, Processing, characteristic composition, absorption features, and principal anti-obesity considerations across major *Camellia sinensis* tea types (As shown in Table 1).

**Table 1:** Processing, characteristic composition, absorption features, and principal anti-obesity considerations across major *Camellia sinensis* tea types.

Tea Type	Processing/Oxidation	Characteristic Constituents	Catechins/EGCG	Oxidized Pigments	Caffeine	Bioavailability/Absorption	Main Interpretation and Limitation
White	Withering and drying; minimal oxidation	Native catechins, caffeine, amino acids	High; EGCG generally high	Very low TF/TR/TB	Moderate	Limited human pharmacokinetic data; parent catechins remain poorly absorbed	Anti-adipogenic and lipolytic effects demonstrated in vitro; controlled human efficacy remains limited [20,21,22]
Green	Heat fixation; non-oxidized	EGCG and other catechins, caffeine, theanine	Highest and best characterized	Trace	Moderate	Rapid but low systemic catechin exposure; extensive conjugation	Best-characterized human exposure and most extensive efficacy literature; effects generally small and heterogeneous [5,9]
Oolong	Partial oxidation	Residual catechins, caffeine, theaflavins and aroma products	Intermediate and variable	Low–moderate TF; low TR	Moderate	Mixed native and oxidized polyphenols; few direct absorption studies	Acute fat-oxidation signal; longer-term evidence limited [23]
Black	Full enzymatic oxidation	Theaflavins, thearubigins, caffeine	Low native catechins	High TF/TR; no characteristic TB dominance	Moderate	Low systemic TF exposure; substantial intestinal residence	Digestive-enzyme and microbiota pathways are plausible; human weight-loss evidence remains limited [17,18,19]
Dark/post-fermented	Microbial pile fermentation or aging	Theabrownins, transformed catechins, polysaccharides, microbial metabolites	Low and product-specific	Variable TR/TB; TB often prominent	Variable	Local intestinal and microbial metabolism may predominate	Bile-acid and microbiota pathways are promising; products are highly heterogeneous [16,24]

Abbreviations: EGCG, epigallocatechin-3-gallate; TF, theaflavins; TR, thearubigins; TB, theabrownins. The descriptors “high”, “moderate”, and “low” indicate relative profiles rather than fixed concentration ranges. Cross-study numerical values are not directly comparable because cultivar, leaf grade, processing, storage, extraction, serving size, and analytical method differ substantially. Future comparative trials should report measured total catechins, EGCG, TF, TR, TB, and caffeine per prepared serving.

### 2.5. Physiologically Achievable Exposure and the Translational Gap

A central translational problem is the mismatch between experimental and human exposure. Many adipocyte studies use 25–100 µM EGCG or total polyphenol concentrations, while plasma concentrations of intact EGCG after oral ingestion are usually much lower [9,10]. Rodent studies also frequently administer extract doses that would correspond to impractical human intakes after body-surface-area scaling. Such studies remain valuable for identifying targets, but they should be interpreted as proof of biological possibility rather than evidence that ordinary tea consumption reproduces the same intracellular concentration.

Three complementary exposure domains should therefore be reported: (i) the concentration of compounds in the prepared beverage or extract; (ii) gastrointestinal exposure, where relatively high local concentrations can influence enzymes, micelles, epithelial cells, and microbes; and (iii) circulating parent compounds and metabolites. Future cell experiments should include low-micromolar concentrations, relevant conjugates, and microbial metabolites, while animal studies should report the extract composition and human-equivalent dose. These practices would reduce overstatement and clarify which mechanisms are compatible with brewed tea, standardized extract, or pharmacological delivery. Representative exposure anchors should therefore be interpreted by evidence level rather than combined into a single “effective dose.” The white-tea adipocyte study used a concentrated extract and included 50 µM EGCG in one component analysis [22]; an acute human crossover study tested 100 or 150 mg of oolong-tea-derived polymerized polyphenols [25]; a 10-week trial administered 200 mL/day of green-tea kombucha [26]; and a six-month black-tea trial used three cups/day [27]. Animal studies in this field use heterogeneous extracts, routes, units, and durations and often do not report sufficient compositional detail for a defensible cross-study dose range. Accordingly, this review does not assign a universal in vitro, animal, or human “effective dose”; exact exposure and formulation should be interpreted within each study.

## 3. Cellular and Metabolic Mechanisms

### 3.1. Inhibition of Digestive Enzymes and Nutrient Absorption

**Figure 1:**
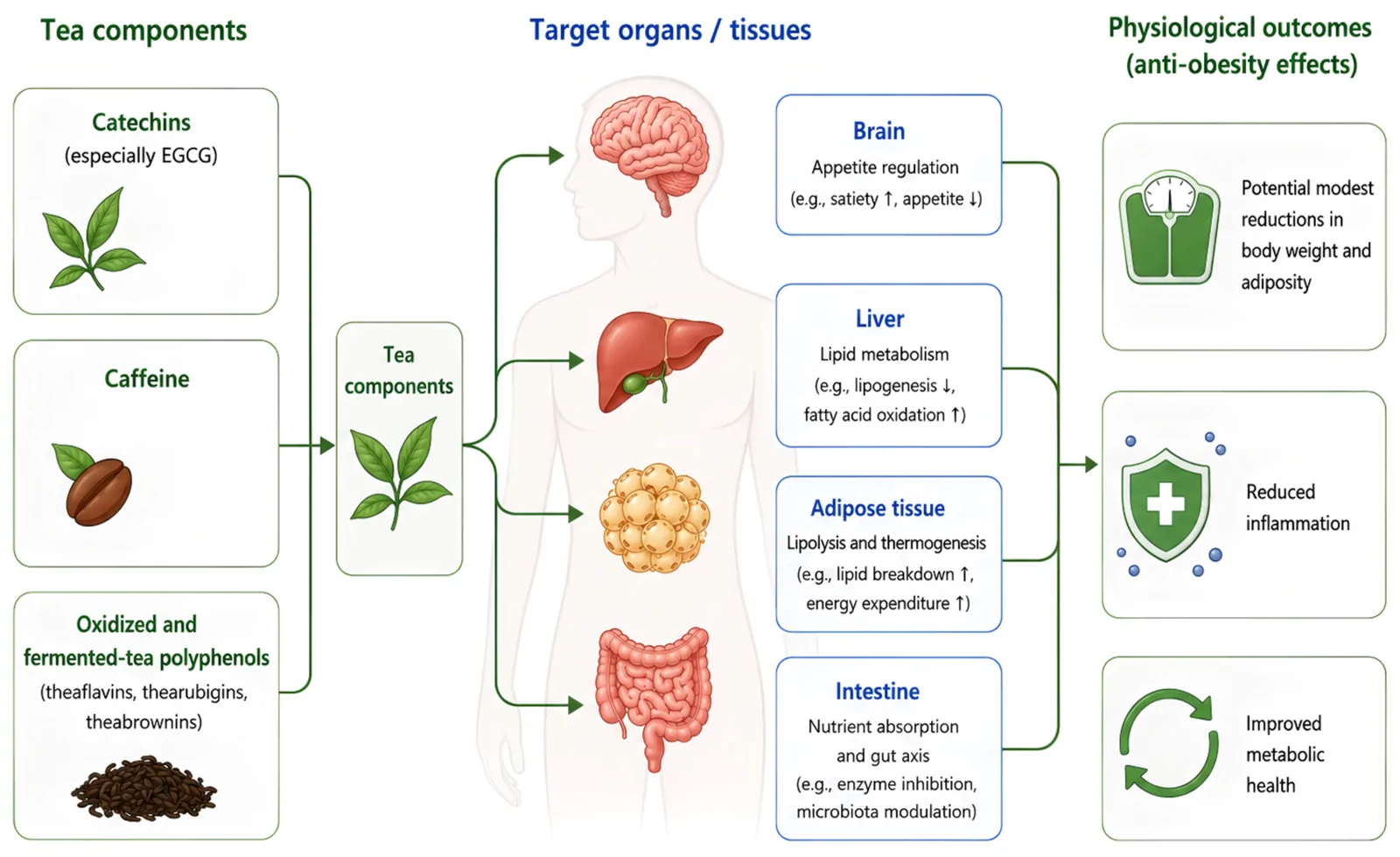
Overview of the proposed physiological targets through which tea bioactive compounds may support obesity management.

Tea polyphenols can reduce the hydrolysis of dietary triglycerides and carbohydrates by interacting with pancreatic lipase, α-amylase, and α-glucosidase. Theaflavins inhibit pancreatic lipase through structure-dependent interactions supported by enzymology and molecular modeling [28], while EGCG can alter α-glucosidase conformation and catalytic activity [29]. Fermentation-related changes in Qingzhuan tea also modify lipase and α-amylase inhibition, illustrating that processing can strengthen or weaken activity depending on the compounds generated [30]. Purple *Camellia sinensis* tea rich in anthocyanins and polyphenols has likewise reduced lipid digestion in experimental systems [31]. The principal organ- and tissue-level targets and potential physiological outcomes of tea bioactive compounds are summarized in Figure 1.

**Figure 2:**
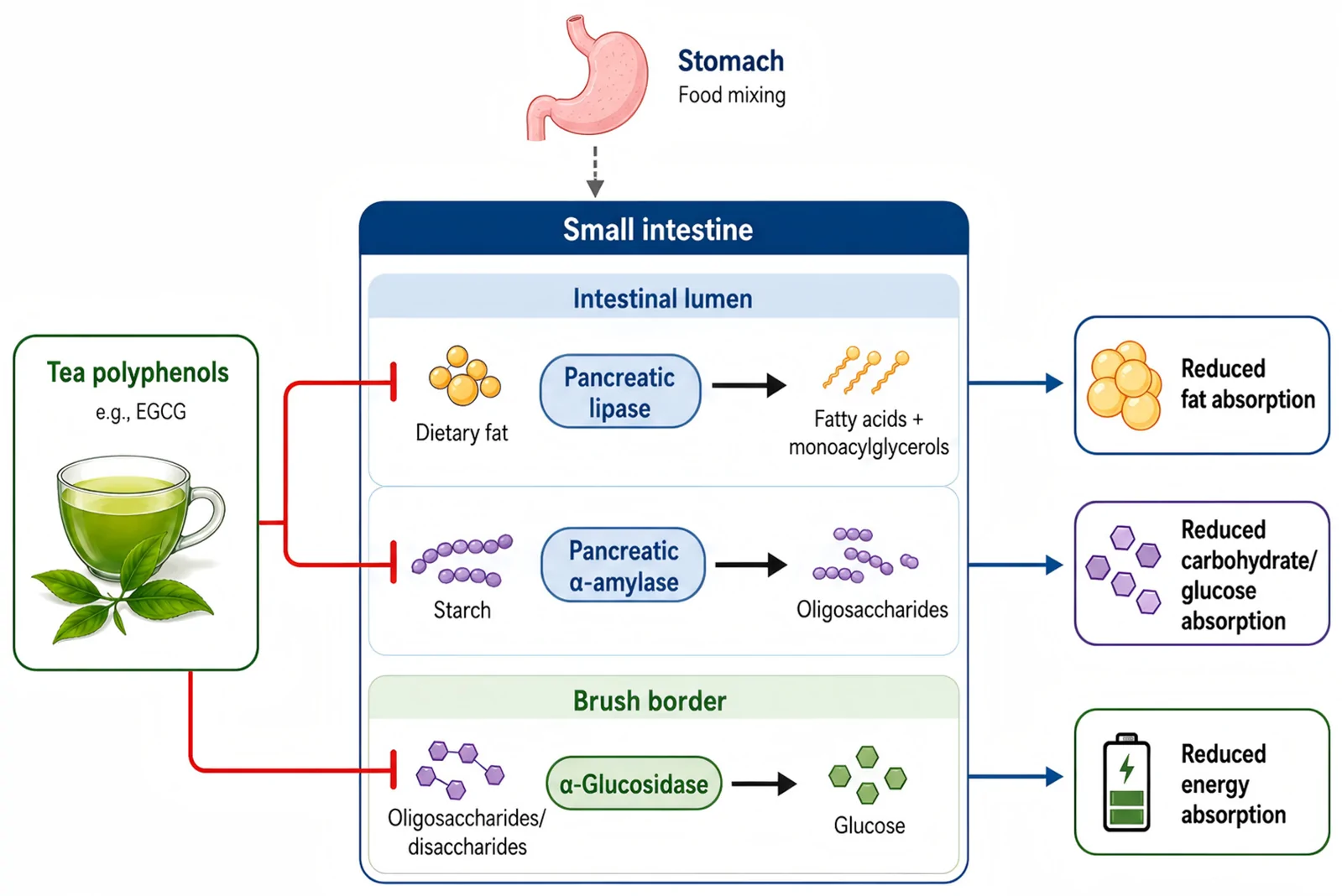
Proposed mechanisms by which tea polyphenols inhibit intestinal digestive enzymes and may reduce fat and carbohydrate absorption.

These actions are most plausible in the intestinal lumen, where compound concentrations exceed those in plasma. However, in vitro enzyme inhibition does not directly establish reduced energy absorption in humans. Protein binding, pH, food emulsions, bile salts, meal composition, and intestinal transit can modify activity. Human studies should therefore combine standardized meal tests with fecal fat, postprandial triglyceride, glucose, and incretin measurements rather than extrapolating from isolated-enzyme assays. A randomized, double-blinded crossover trial in 50 healthy adults found that oolong-tea-derived polymerized polyphenols at doses up to 150 mg did not clearly reduce early postprandial triacylglycerol responses, although their combination with caffeine and catechins transiently lowered glucose and insulin concentrations [25]. This finding reinforces the distinction between digestive-enzyme inhibition in vitro and clinically detectable effects after a mixed meal. The proposed intestinal mechanisms linking digestive-enzyme inhibition to reduced nutrient absorption are summarized in Figure 2.

### 3.2. AMPK Signaling, Fatty-Acid Oxidation, and Lipogenesis

AMP-activated protein kinase (AMPK) integrates cellular energy status with lipid synthesis and oxidation. Experimental tea interventions increase AMPK phosphorylation, inhibit acetyl-CoA carboxylase (ACC), reduce sterol regulatory element-binding protein 1c (SREBP-1c) and fatty-acid synthase, and facilitate carnitine-dependent fatty-acid entry into mitochondria. Tea catechins have also stimulated hepatic lipid catabolism and reduced diet-induced lipid accumulation [32,33]. These changes provide a coherent mechanism for reduced hepatic steatosis and adiposity in rodents.

Adipogenesis is additionally regulated through PPARγ and C/EBPα. EGCG and theaflavins can suppress these transcriptional programs in preadipocytes and obese rodents, whereas caffeine increases cyclic adenosine monophosphate (cAMP) and hormone-sensitive lipase (HSL) signaling. Because mature human adipose tissue is not equivalent to 3T3-L1 cells, clinical studies should prioritize adipose-tissue or circulating metabolic biomarkers and avoid presenting cell-line effects as established human mechanisms.

### 3.3. Thermogenesis and Adipose-Tissue Remodeling

Green-tea extracts and catechins can promote brown-adipose-tissue activity or beige remodeling of white adipose tissue in rodents. Reported changes include increased uncoupling protein 1 (UCP1), peroxisome proliferator-activated receptor γ coactivator 1α, mitochondrial biogenesis, and adiponectin-dependent thermogenic signaling [34,35,36,37]. Caffeine may reinforce these effects through sympathetic activation and norepinephrine release.

**Figure 3:**
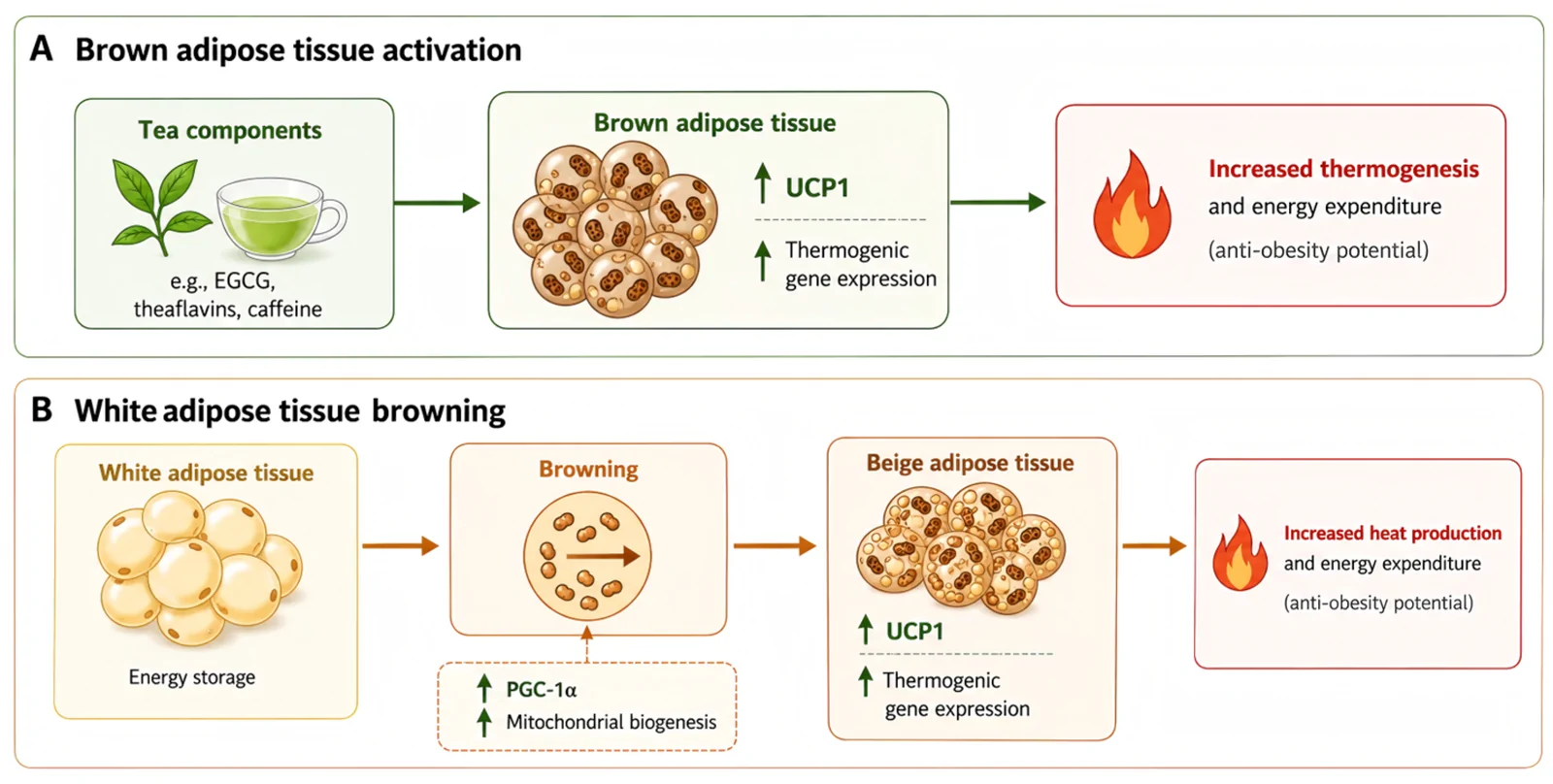
Proposed mechanisms by which tea components may activate brown adipose tissue and promote white-adipose-tissue browning, thereby increasing thermogenesis and energy expenditure.

Translation to humans is uncertain. Human brown-fat mass and activation vary with age, adiposity, ambient temperature, diet, and previous cold exposure. Small increases in 24 h energy expenditure may not accumulate into meaningful weight loss if appetite or intake compensates. Thus, thermogenesis should be considered a biologically plausible contributor rather than a sufficient explanation for clinical efficacy [38]. The proposed activation of brown adipose tissue and browning of white adipose tissue are illustrated in Figure 3.

### 3.4. Integrated and Compound-Specific Contributions

**Figure 4:**
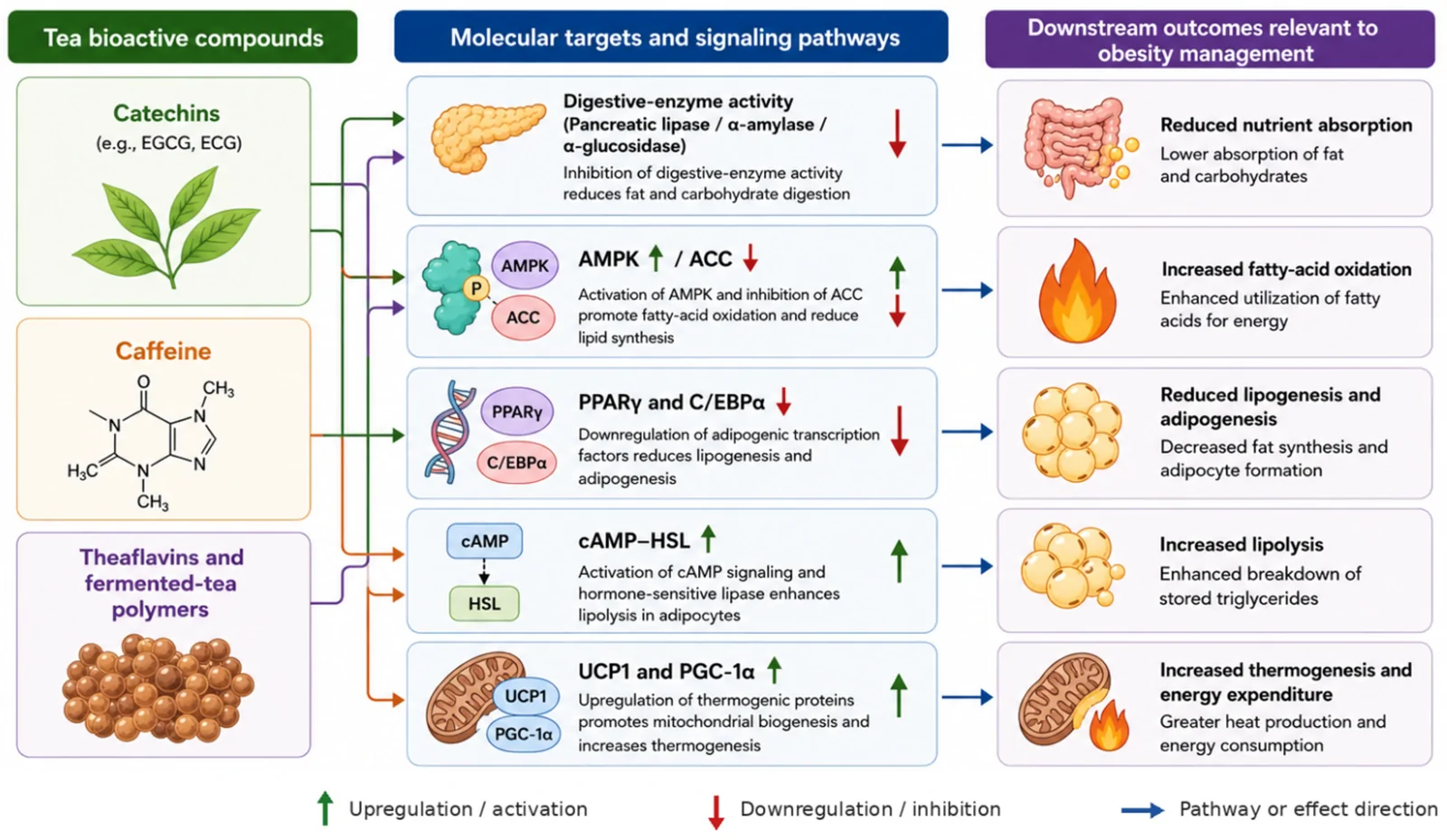
Integrated molecular targets and signaling pathways through which tea bioactive compounds may support obesity management.

The major mechanism axes are complementary. Catechins contribute most clearly to enzyme interaction, AMPK-related lipid handling, and microbiota substrates; caffeine provides acute neural and lipolytic stimulation; and theaflavins or larger fermented tea polymers contribute intestinal enzyme inhibition and microbiota-dependent effects. Synergy is plausible when components act at different sites, but “whole-tea synergy” should be demonstrated rather than assumed. Factorial trials comparing isolated components, reconstituted mixtures, and matched brewed tea are needed to separate additive effects from true interactions. The compound-specific molecular targets, signaling pathways, and downstream outcomes are integrated in Figure 4. Principal tea bioactive compounds, molecular targets, downstream outcomes, and evidence level, (As shown in Table 2).

**Table 2:** Principal tea bioactive compounds, molecular targets, downstream outcomes, and evidence level.

Compound/Class	Representative Targets	Downstream Outcome	Current Evidence Interpretation
EGCG and other catechins	Pancreatic lipase; α-glucosidase; AMPK; ACC; SREBP-1c; PPARγ/C/EBPα	↓ nutrient hydrolysis; ↑ fatty-acid oxidation; ↓ lipogenesis/adipogenesis	Cell/rodent: strong; human mechanistic confirmation: limited [29,32]
Caffeine	Adenosine receptors; sympathetic/catecholamine signaling; cAMP–HSL	Acute lipolysis and thermogenic stimulation	Acute human physiology supported; chronic weight effect inconsistent [11]
Theaflavins	Pancreatic lipase; AMPK-related pathways; inflammatory signaling	↓ fat digestion; improved glucose/lipid handling in rodents	Enzyme/rodent evidence; low systemic exposure [17,28]
Thearubigins/theabrownins	Microbial metabolism; bile acids; intestinal barrier	Local intestinal effects; altered microbial metabolites and host signaling	Promising animal evidence; composition and human exposure poorly standardized [19,24]
Tea polysaccharides and mixed extracts	Microbiota; barrier proteins; inflammatory mediators	SCFA production, barrier reinforcement, reduced endotoxemia	Predominantly animal evidence; active structures incompletely defined [39,40]

Abbreviations: ACC, acetyl-CoA carboxylase; AMPK, AMP-activated protein kinase; cAMP, cyclic adenosine monophosphate; C/EBPα, CCAAT/enhancer-binding protein α; EGCG, epigallocatechin-3-gallate; HSL, hormone-sensitive lipase; PPARγ, peroxisome proliferator-activated receptor γ; SCFA, short-chain fatty acid; SREBP-1c, sterol regulatory element-binding protein 1c. Arrows indicate an increase or decrease.

## 4. Gut Microbiota, Microbial Metabolites, and the Gut–Liver Axis

### 4.1. Taxon- and Function-Specific Microbiota Responses

A substantial fraction of tea polyphenols reaches the colon, where they can alter microbial growth and are converted into phenyl-γ-valerolactones, phenylvaleric acids, and other low-molecular-weight metabolites. Animal studies commonly report shifts in *Akkermansia*, *Bifidobacterium*, or selected short-chain fatty acid-producing taxa and reductions in pathobiont or endotoxin-associated organisms, but the direction of change is not universal [39,40,41,42]. Where species-level resolution was available, *Akkermansia muciniphila* increased after Fu brick tea polyphenol or EGCG interventions and was associated with improved obesity-related phenotypes [39,42]. Other studies reported only genus-level signals—for example, *Enterococcus*, *Intestinimonas*, *Blautia*, *Bilophila*, *Bacteroides*, and *Akkermansia*—and these should not be converted into species-level claims; the relevant taxa and source resolution are summarized in Table 3. Firmicutes-to-Bacteroidota ratios should not be interpreted as stand-alone markers of health because this aggregate metric is sensitive to methods, diet, and baseline community structure.

Fu brick tea polyphenols have improved intestinal oxidative stress and barrier function in obese mice, and tea-polyphenol interventions have linked microbial shifts with altered short-chain fatty acid (SCFA) profiles, tighter barrier proteins, and reduced inflammation [40]. EGCG may also protect the ileal barrier through hypoxia-inducible factor-1*α* (*Hif1a*)-associated signaling and specific microbial changes [47]. Black tea, oolong tea, and raw or ripened Pu-erh tea produce distinct microbial responses, emphasizing that “beneficial bacteria” should be replaced by named taxa, functions, and metabolites [44,45,46].

### 4.2. SCFAs, Phenolic Metabolites, and Intestinal Barrier Function

SCFAs—particularly acetate, propionate, and butyrate—can influence colonocyte energy supply, barrier integrity, enteroendocrine signaling, and hepatic substrate metabolism. Tea-associated changes in SCFA profiles may result from enrichment of fermentative organisms or cross-feeding, although greater fecal SCFA concentration can reflect either increased production or reduced absorption. Microbial catechin metabolites may circulate at higher concentrations and for longer periods than intact parent catechins, providing a potential bridge between low parent-compound bioavailability and systemic effects [40,43].

Human evidence remains less consistent than rodent evidence. Small studies demonstrate that green tea can alter the human microbiome, but responses depend on baseline diet, microbial community, antibiotic exposure, and analytical pipeline [43]. Trials should prespecify taxonomic and functional outcomes, control diet, quantify polyphenol metabolites, and report absolute as well as relative abundance. In a 10-week randomized trial conducted during energy restriction, 200 mL/day of green-tea kombucha improved selected gastrointestinal symptoms and altered the serum metabolome, but did not significantly change gut-microbiota composition or intestinal permeability outcomes relative to the control group after adjustment [26]. These findings illustrate that a fermented tea beverage may influence host responses without producing a consistent taxonomic microbiome signal.

### 4.3. Bile-Acid Metabolism and Gut–Liver Endocrine Signaling

Bile acids are not only detergents but also endocrine signals. Microbial bile salt hydrolases (BSHs) and downstream transformations shape the intestinal bile-acid pool, which can signal through the farnesoid X receptor (FXR), fibroblast growth factor 15/19, and Takeda G-protein-coupled receptor 5 (TGR5). Unabsorbed tea polyphenols and polymers can modify bile-acid deconjugation, fecal excretion, and microbial composition. Theabrownin from Pu-erh tea reduced microbial BSH activity and altered the FXR–FGF15 axis in experimental models, providing a mechanistic link between post-fermented tea, cholesterol metabolism, and the gut–liver axis [24]. EGCG–caffeine combinations have also affected bile-acid metabolism in obese mice [12]. Green-tea polyphenols have also modified the gut-microbiota-dependent metabolism of energy, bile constituents, and micronutrients in rats, providing additional experimental support for this microbiota–bile-acid pathway [48].

This pathway should be evaluated with targeted bile-acid profiling rather than inferred solely from microbiota composition. Human trials need paired measurements of serum and fecal bile acids, FGF19, lipid outcomes, and microbial bile-acid genes. Because FXR signaling has tissue-specific and context-dependent effects, simplified claims that one direction of FXR modulation is universally beneficial should be avoided.

## 5. Appetite, Endocrine Signaling, and Inflammation

### 5.1. Appetite-Related Hormones

Potential appetite pathways include glucagon-like peptide-1 (GLP-1), peptide YY (PYY), ghrelin, cholecystokinin, and central catecholamine signaling. Tea polyphenols may influence enteroendocrine cells indirectly through short-chain fatty acids, bile acids, postprandial glucose, or intestinal nutrient delivery, while caffeine may transiently suppress perceived appetite in some individuals. Animal evidence includes an EGCG model in which bile-acid/TGR5 signaling and enrichment of *Akkermansia muciniphila* were accompanied by increased GLP-1 release and serum PYY [42]. Previous reviews describe heterogeneous and generally limited human evidence for tea-related appetite and endocrine pathways [49,50]. A GRADE-assessed meta-analysis of green-tea extract trials found no overall effect on ghrelin or leptin but reported increased adiponectin; direct standardized GLP-1 and PYY measurements remain sparse [51]. For oolong, black, and dark tea, no adequately standardized direct hormone evidence was identified. Appetite suppression should therefore not be presented as a clinically established weight-loss mechanism.

### 5.2. Inflammation and Adipokines

Obesity-associated inflammation involves adipose macrophages, tumor necrosis factor α, interleukin-6, nuclear factor kappa B (NF-κB), lipopolysaccharide signaling, and altered adiponectin or leptin. Tea interventions can reduce inflammatory markers and improve barrier integrity in rodents, but effects in humans are generally modest and may reflect concurrent changes in body weight, diet quality, or insulin sensitivity [39,40,44,47,49,51]. Trials should distinguish direct anti-inflammatory actions from secondary improvement caused by energy restriction or fat loss, Evidence regarding the relationship between the main types of tea and appetite-regulating hormones and inflammatory signalling pathways, as shown in Table 4.

**Table 4:** Evidence relating major tea types to appetite hormones and inflammatory signaling.

Tea Type	Study Model	Observed/Putative Hormone Effect	Proposed Mechanism	Interpretive Limitation
Green tea/catechins	Rodent mechanistic studies and human RCT/meta-analysis	Mouse EGCG model: ↑ GLP-1 and PYY; pooled human GTE evidence: ↑ adiponectin, no overall ghrelin or leptin effect	Bile acid–TGR5 signaling, *A. muciniphila*, SCFAs, delayed nutrient absorption	Human GLP-1/PYY data remain sparse; hormone changes do not establish reduced energy intake [42,51]
Oolong tea	Animal and acute human metabolic studies	No standardized direct GLP-1/PYY/ghrelin outcome identified	Partial oxidation products and caffeine; indirect pathways remain hypothetical	Evidence gap; cited oolong studies focus on fat oxidation, adiposity, or microbiota rather than appetite hormones [23,45,52]
Black tea/theaflavins	Animal and mechanistic studies	No direct standardized appetite-hormone endpoint identified	Digestive-enzyme, microbial, and inflammatory pathways	Human meal-challenge studies are needed; existing evidence does not establish GLP-1/PYY/ghrelin effects [17,44]
Dark tea/theabrownins	Animal and limited clinical	Indirect endocrine effects through bile acids are plausible; direct hormone data are absent	BSH–FXR–FGF15/19–TGR5 and microbial metabolites	Human endocrine validation lacking [24,46]
Across tea types	Mixed	Inflammatory markers and adiponectin may improve, but effect size and causality vary	NF-κB, endotoxemia, adipokines, weight change	Control diet, weight change, and baseline inflammation; evidence is mainly experimental or indirect [39,40,44,47,49,51]

Abbreviations: BSH, bile salt hydrolase; FGF15/19, fibroblast growth factor 15/19; FXR, farnesoid X receptor; GLP-1, glucagon-like peptide-1; GTE, green-tea extract; NF-κB, nuclear factor kappa B; PYY, peptide YY; RCT, randomized controlled trial; SCFA, short-chain fatty acid; TGR5, Takeda G-protein-coupled receptor 5. Arrows indicate an increase or decrease. “No direct endpoint identified” denotes an evidence gap in the cited tea-specific efficacy studies, not proof of no biological effect.

## 6. Comparative Efficacy and Determinants of Response

### 6.1. Comparative Interpretation Across Tea Types

Green tea has the most extensive human evidence because its catechin content is relatively well characterized and standardized extracts are widely available. This does not prove biological superiority, but it permits more precise estimates. Oolong tea combines residual catechins, caffeine, and oxidation products; short-term crossover studies report increased fat oxidation, but longer trials remain limited [23,52]. Black and dark teas may rely more strongly on local intestinal, microbial, and bile-acid pathways. Direct head-to-head trials matching caffeine, energy intake, and total polyphenols are rare, so definitive statements that one tea type produces the strongest or most durable weight loss are premature. Direct human evidence for oxidized and post-fermented teas is emerging but remains limited. In a six-month double-blind trial, three cups/day of black tea produced small improvements in body weight and waist measures at three months, but these differences were not sustained at six months [27]. A 90-day randomized study of Liupao dark tea in 106 adults with overweight or obesity reported within-group reductions in body weight, adiposity measures, blood pressure, and selected lipids; however, the absence of a non-tea placebo and the lack of clear differences among tea-aging groups limit causal interpretation [53].

### 6.2. Dose–Response and Safety

Dose–response relationships are not linear. Higher extract doses may increase gastrointestinal exposure but can also reduce tolerability and amplify caffeine-related symptoms or liver risk. No reproducible efficacy plateau or minimum effective EGCG dose has been established because trials differ in product mass, EGCG and caffeine content, formulation, duration, fed or fasted administration, and participant characteristics. Nominal extract-dose subgroup findings should therefore not be converted into a universal dosing recommendation. The European Food Safety Authority concluded that catechins from traditional green-tea infusions are generally not associated with safety concerns, whereas supplemental EGCG doses at or above 800 mg/day were associated with statistically significant elevations in serum transaminases; a no-effect threshold could not be established [54]. The 800 mg value should therefore not be misrepresented as a safe upper limit.

Safety assessment should distinguish brewed tea from concentrated extracts, specify fasting or fed administration, and record total EGCG, total catechins, caffeine, co-medications, alcohol use, and pre-existing liver disease. Recent reviews emphasize liver-related risk with concentrated EGCG preparations, whereas a defined decaffeinated green-tea-polyphenol intervention in girls with obesity reported no major adverse health effects under trial conditions [55,56]. Tea consumed with an iron-containing meal can substantially inhibit non-heme iron absorption; a controlled stable-isotope trial showed that separating tea from the meal by 1 h attenuated this effect [57]. Population-wide advice should therefore favor moderate unsweetened tea intake and avoid unsupervised high-dose extracts, particularly during fasting or in people with liver risk or iron deficiency.

### 6.3. Brewing Conditions and the Food Matrix

Water temperature, infusion time, leaf particle size, agitation, leaf-to-water ratio, and repeated infusion determine the amount and pattern of caffeine and polyphenols released. Hotter water and longer extraction generally accelerate release but may also increase bitterness, astringency, oxidation, or epimerization. Controlled extraction studies demonstrate distinct time–temperature optima for individual catechins and caffeine rather than a single universally optimal brew [58,59]. Cold brewing slows extraction and changes the relative composition; it should not be described simply as “lower” or “better” without reporting time and dose.

Food-matrix interactions are similarly nuanced. Milk proteins bind catechins in vitro and can change bioaccessibility, digestibility, and antioxidant assays [60], yet a randomized crossover study found that adding milk to black tea did not significantly reduce plasma catechin exposure [61]. Sugar and caloric creamers can increase beverage energy and postprandial glycemia, potentially offsetting the value of replacing energy-dense drinks with unsweetened tea. Future trials should report the actual prepared beverage, not only the dry leaf or capsule label.

### 6.4. Interindividual Variability

Response heterogeneity may arise from catechol-O-methyltransferase and caffeine-metabolism genotypes, habitual caffeine use, sex, age, menopausal status, adiposity, insulin resistance, baseline diet, sleep, physical activity, and gut-microbial capacity to transform polyphenols. Obesity itself can alter catechin pharmacokinetics [9]. Trials should prespecify plausible modifiers, avoid underpowered post hoc subgroup claims, and collect adherence biomarkers when possible.

## 7. Human Clinical Evidence and Clinical Meaning

### 7.1. Meta-Analyses and Evidence Quality

Clinical syntheses do not support large or uniform weight loss. A Cochrane review concluded that green-tea preparations produced small, statistically non-significant weight loss in studies conducted outside Japan (i.e., in non-Japanese populations or settings) and were unlikely to be clinically important [62]. A more recent time- and dose–response meta-analysis in women with overweight or obesity reported weighted mean differences (WMDs) of −1.23 kg for body weight, −0.47 kg/m^2^ for body mass index (BMI), and −3.46 cm for waist circumference. Heterogeneity was outcome-dependent (I^2^ = 42% for body weight, 65% for BMI, and 75% for waist circumference), reflecting differences in dose, duration, product, caffeine content, population, and trial methods [63]. Meta-analyses also suggest modest improvements in selected lipid and blood-pressure outcomes [64].

Statistical significance should not be equated with clinical importance. A 1–2 kg average reduction is substantially below the ≥5% body-weight loss commonly used as a benchmark for clinically meaningful metabolic benefit in obesity care, although small changes in waist circumference, triglycerides, or beverage substitution may still be relevant at the population level. Interpretation is weakened by short duration, small samples, inconsistent comparators, imperfect blinding due to taste or caffeine effects, selective reporting, and outcome heterogeneity. Funnel plots and regression-based publication-bias tests are underpowered when only a few trials are available; therefore, a non-significant test should not be interpreted as proof that publication bias is absent.

### 7.2. Representative Human Trials

Individual trials illustrate both promise and uncertainty. Decaffeinated green-tea polyphenols have been evaluated in girls with obesity, with reported body-composition outcomes and no major safety signal in the trial setting [56,65]. A pilot intervention in adolescents reported associations between tea or coffee consumption and weight-control outcomes, but sample size and intervention complexity limit inference [66]. White-tea and Pu-erh-tea studies report improvements in anthropometric or lipid variables, yet independent replication, product standardization, and adequate blinding remain priorities [21,67]. Recent human studies therefore broaden tea-type coverage without resolving the central uncertainty: acute oolong-polyphenol effects were endpoint-specific [25], green-tea kombucha produced gastrointestinal and metabolomic signals without a robust microbiome effect [26], and black or Liupao tea studies reported modest or methodologically constrained anthropometric improvements [27,53].

The best-supported conclusion is therefore not that tea “treats obesity,” but that certain preparations may produce small adjunctive effects in selected populations. Clinicians should evaluate tea within the patient’s total dietary pattern, caffeine tolerance, liver risk, iron status, medications, and treatment goals. Researchers should report CONSORT-relevant methods, intervention composition, adherence, adverse events, and clinically interpretable outcomes, Representative clinical reviews and trials relating to tea and obesity-related outcomes. As shown in Table 5.

**Table 5:** Representative clinical syntheses and trials relevant to tea and obesity-related outcomes.

Study	Design/Population	Intervention	Main Finding	Source-Reported Quality/Certainty and Interpretive Issue
Jurgens et al., 2012 [62]	Cochrane review of RCTs	Green-tea preparations; variable dose/duration	Small, non-significant weight loss in non-Japanese studies; no clear maintenance effect	Formal Cochrane risk-of-bias assessment; source judged effects unlikely clinically important; no single GRADE category was reported [62]
Zhang et al., 2024 [63]	15 articles; overweight/obese women	Green-tea supplementation; variable catechin doses; mostly ≥several weeks	WMD: −1.23 kg body weight (95% CI −2.13 to −0.33); −0.47 kg/m^2^ BMI (−0.87 to −0.07); −3.46 cm waist (−6.75 to −0.16)	No formal GRADE certainty reported; I^2^ = 42% (weight), 65% (BMI), and 75% (waist), with variable products, doses, populations, and methods [63]
Asbaghi et al., 2024 [51]	GRADE-assessed systematic review/meta-analysis; 59 studies, 3802 participants	Green-tea extract; variable dose and duration	Reduced body mass, BMI, and body-fat percentage; increased adiponectin; no overall effect on leptin or ghrelin	GRADE certainty ranged from low to high across outcomes; formulation and outcome heterogeneity remain [51]
Akyildiz et al., 2024 [21]	Adults with obesity	White tea; intervention-specific preparation	Improved selected anthropometric/metabolic measures	Single trial; no formal evidence-certainty grade; independent blinded replication and compositional verification are needed [21]
Xie et al., 2021 [65]	Girls with obesity; RCT	Decaffeinated green-tea polyphenols	Body-fat and endocrine outcomes evaluated	Population-specific RCT; no formal evidence-certainty grade; generalizability and long-term benefit remain uncertain [65]
Jensen et al., 2016 [67]	Hyperlipidemic adults; randomized placebo-controlled	Pu-erh tea extract; 20 weeks	Reduced selected body-fat and lipid outcomes	Single product and sponsor context; no formal evidence-certainty grade; independent replication is needed [67]
Gholami et al., 2024 [68]	10 RCTs; exercise participants with overweight/obesity	Green-tea catechins plus exercise vs. exercise control	Small additional effects: weight SMD −0.30, BMI −0.33, and fat mass −0.29; I^2^ = 0% for all three	No formal GRADE certainty reported; no detected publication bias for body-composition outcomes, but lipid outcomes were null and heterogeneous (I^2^ = 53–91%) [68]
Perkin et al., 2023 [25]	50 healthy adults; randomized, double-blinded crossover	Oolong-tea-derived polymerized polyphenols (100 or 150 mg, single acute dose; not per kg body weight or per kg diet), with or without caffeine/catechins	No clear reduction in early postprandial triacylglycerol; the combined formulation transiently lowered glucose and insulin	Acute healthy-adult meal-challenge model; does not establish chronic weight or adiposity effects [25]
Bøhn et al., 2014 [27]	111 adults; double-blind, placebo-controlled 6-month trial	Three cups/day black tea vs. flavonoid-free, caffeine-matched placebo	Small reductions in weight gain and waist measures at 3 months; effects were not sustained at 6 months	Direct black-tea evidence, but the effect was modest and transient [27]
Wang et al., 2025 [53]	106 adults with overweight/obesity; randomized, double-blind 90-day study	Liupao tea aged 1, 4, 7, or 10 years	Within-group improvements in body composition, blood pressure, and selected lipids; no clear aging-duration superiority	No non-tea placebo; between-group evidence does not isolate the tea effect [53]

Abbreviations: BMI, body mass index; CI, confidence interval; GRADE, Grading of Recommendations Assessment, Development and Evaluation; I^2^, heterogeneity statistic; RCT, randomized controlled trial; SMD, standardized mean difference; WMD, weighted mean difference. “Not formally graded” means that the source did not report a comparable evidence-certainty category; it does not imply high quality. Percentage change in body weight was not calculated when the source did not explicitly report it or when compatible baseline and follow-up data were unavailable; pooled WMDs and SMDs were not converted to percentages.

## 8. Tea Combined with Dietary and Exercise Strategies

### 8.1. Dietary Context

The clearest practical role for tea is as an unsweetened replacement for sugar-sweetened or energy-dense beverages. In polyphenol-rich dietary patterns, tea may contribute to a broader food matrix rather than act as the sole active agent. The DIRECT PLUS trial showed that a green-Mediterranean diet enriched with green plants and polyphenols, combined with physical activity, produced greater reductions in visceral and intrahepatic fat than comparison dietary strategies [69,70]. These findings support a polyphenol-rich lifestyle pattern but cannot isolate tea-specific causality because the intervention simultaneously modified multiple foods and behaviors.

### 8.2. Exercise Context

**Figure 5:**
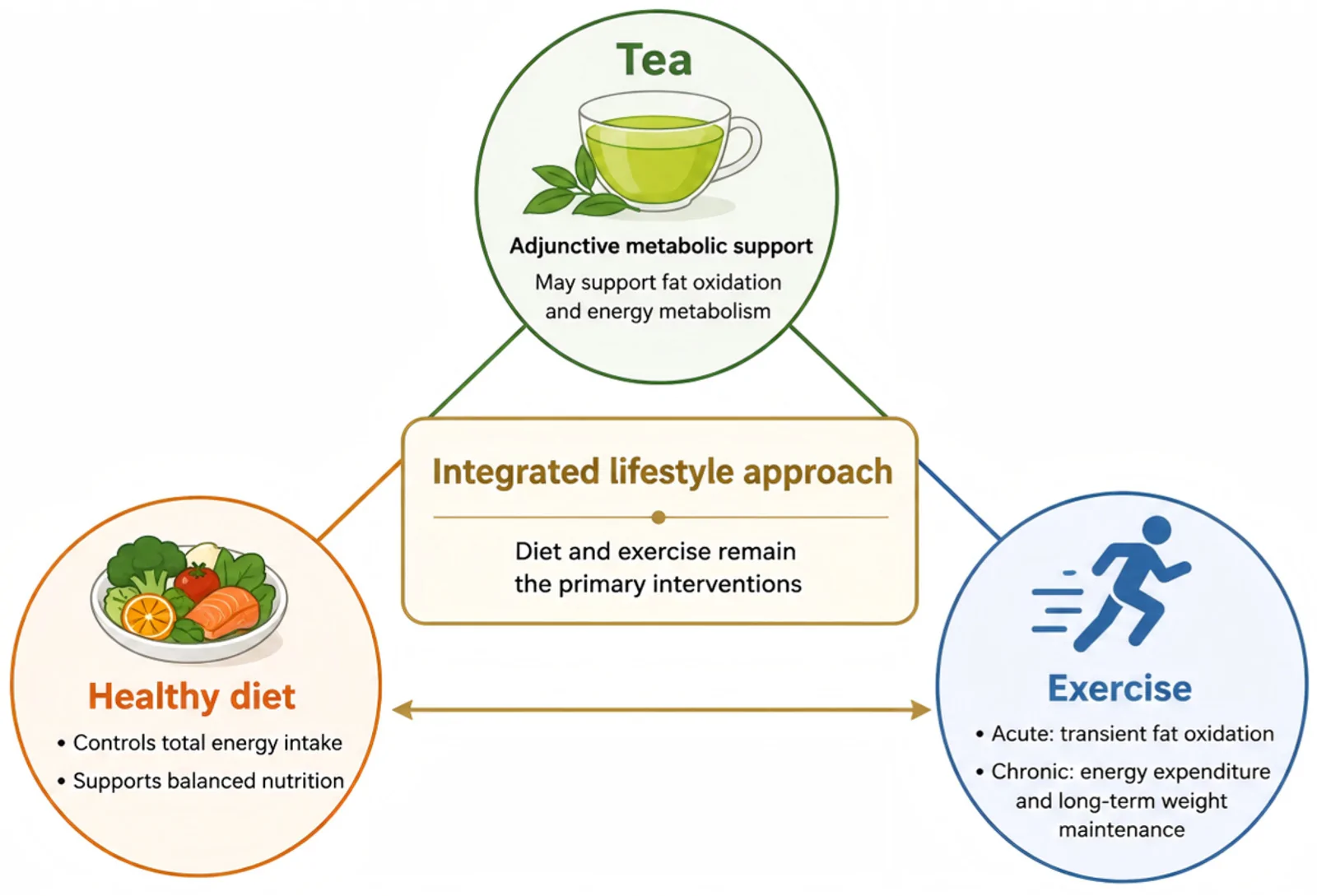
Integrated roles of tea, dietary control, and exercise in obesity management.

Acute pre-exercise caffeine or oolong/green tea may increase fat oxidation under controlled conditions, but acute substrate oxidation does not necessarily predict chronic fat loss. A 2024 meta-analysis of 10 randomized trials found small additional effects of green-tea catechins plus exercise on body weight (standardized mean difference [SMD] −0.30), body mass index (SMD −0.33), and fat mass (SMD −0.29), with I^2^ = 0% for these three body-composition outcomes; lipid outcomes were not significantly improved and showed substantially greater heterogeneity [68]. Exercise prescriptions should therefore be based on established health and fitness benefits, with tea considered optional and secondary. These dietary and exercise contexts are compared in Table 6 and conceptually summarized in Figure 5.

## 9. Future Research Priorities

Future research should prioritize quality over the accumulation of loosely comparable studies. First, interventions must be chemically defined: tea species and cultivar, processing category, batch, brewing protocol, total catechins, EGCG, caffeine, theaflavins, and relevant polymeric fractions should be reported. Second, mechanistic studies should use physiologically attainable exposures and include conjugated or microbiota-derived metabolites. Third, clinical trials should be sufficiently long to evaluate maintenance, use credible caffeine- and sensory-matched comparators where possible, and distinguish statistical from clinical significance.

Microbiome-informed personalization is promising but requires validation across populations and laboratories. Baseline microbial functions, rather than isolated taxonomic markers, may help identify participants who efficiently transform catechins or modify bile acids [71]. Strategies to improve bioavailability can be grouped into four categories: optimization of brewing or extraction to increase release into the consumed beverage [58,59]; food-matrix or co-formulation approaches that modify catechin binding and bioaccessibility [60,61]; encapsulation or nano-delivery to protect catechins and control intestinal release [72,73,74]; and fermentation or biotransformation to generate smaller or more persistent metabolites [75]. These approaches may improve chemical stability, gastrointestinal exposure, or systemic availability, but none should be assumed to increase clinical weight loss without exposure-confirmed human trials. Carrier composition, dose, production organisms, metabolite identity, batch consistency, and safety require separate evaluation.

**Figure 6:**
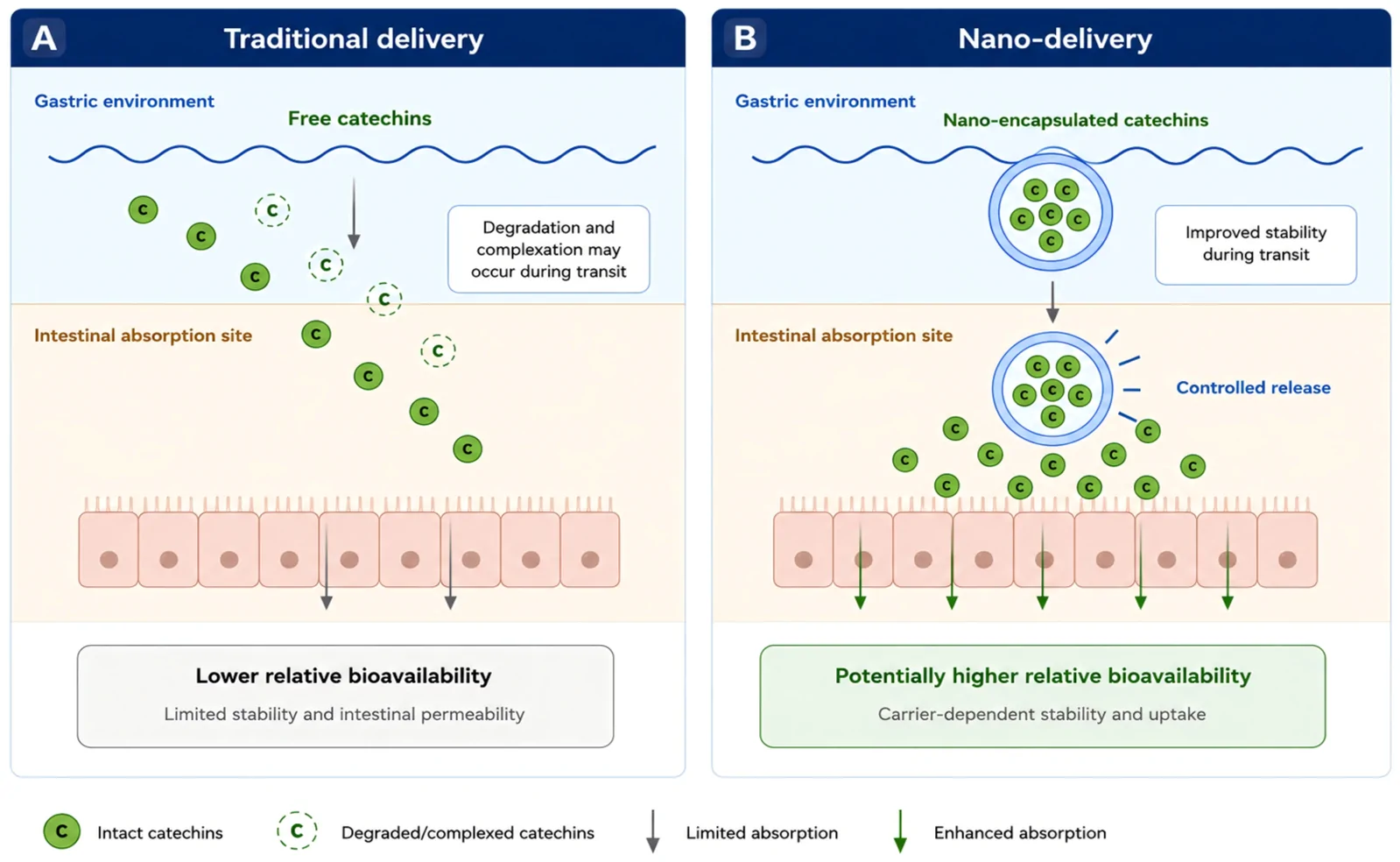
Comparison of traditional and nano-delivery pathways for tea catechins during gastrointestinal transit and intestinal absorption.

A practical research roadmap is: (1) standardized chemistry and brewing; (2) exposure-confirmed mechanistic studies; (3) phase-appropriate safety assessment; (4) adequately powered human trials with body-composition and metabolic endpoints; and (5) independent replication. Shared reporting standards would allow future meta-analyses to compare tea types, doses, and responders more reliably. A comparison of conventional and nanodelivery routes for tea catechins in terms of their passage through the gastrointestinal tract and absorption in the intestine is shown in Figure 6.

## 10. Conclusions

Tea contains a processing-dependent mixture of bioactive compounds capable of influencing digestive enzymes, AMPK-related lipid handling, adipose thermogenesis, intestinal barrier function, microbial metabolites, and bile-acid signaling. Evidence for these pathways is strongest in biochemical, cell, and animal models. In humans, green-tea catechin–caffeine preparations have the most developed evidence base, but average effects on body weight are generally small and heterogeneous—with this heterogeneity primarily derived from the pool of green-tea studies. For oolong, black, and dark teas, available evidence remains too limited in quantity and standardization to permit comparable assessments of heterogeneity or uniform effect magnitude. Meta-analytic estimates of approximately 1–2 kg average weight loss are generally below the ≥5% body-weight reduction commonly used as a clinically meaningful benchmark, and heterogeneity further weakens any expectation of a uniform response. Evidence for white, oolong, black, and dark teas is promising but less standardized and less frequently replicated.

Current evidence does not support tea as a primary treatment for obesity. Unsweetened tea may be used as a culturally acceptable, low-energy adjunct to dietary quality and physical activity, while concentrated extracts require attention to dose, liver safety, caffeine, iron absorption, and product quality. Researchers should align experimental exposure with human physiology, standardize tea composition and preparation, and conduct longer comparative trials. Clinicians should frame tea realistically within established obesity care, and the public should avoid interpreting mechanistic findings or high-dose supplement marketing as proof of substantial weight loss.

## Figures and Tables

**Table 3:** Representative microbiota, metabolite, and host-signaling responses reported for major tea classes.

Tea/Exposure	Microbial Feature	Metabolite/Signaling Mediator	Metabolic Interpretation	Evidence Limitation
Green tea/catechins	*Akkermansia muciniphila* in selected rodent studies; other changes often reported only at genus or functional level	Phenyl-γ-valerolactones; SCFAs; bile-acid/TGR5 signals	Barrier reinforcement, lower endotoxin signaling, and altered hepatic lipid or enteroendocrine signaling	Mostly rodents; small heterogeneous human studies; taxonomic resolution varies [41,42,43]
Fu brick tea	*A. muciniphila*; *Alloprevotella*, *Bacteroides*, and *Faecalibaculum* reported mainly at genus level	SCFAs; barrier and oxidative-stress signals	Improved barrier proteins and inflammatory profile	Rodent evidence; only *A. muciniphila* was resolved to species in the cited study [39]
Black tea	Product-dependent genus-level shifts; microbiota-dependent transfer of the anti-obesity phenotype	Polymer-derived phenolics	Changes in host lipid-related gene expression	Rodent evidence; polymers and taxonomic signals remain incompletely characterized [19,44]
Oolong tea	*Enterococcus*, *Intestinimonas*, *Blautia*, *Bilophila*, and other genera; no species-level resolution	SCFAs and phenolic metabolites proposed	Reduced adiposity and improved lipid handling	Predominantly animal evidence; genus-level associations do not establish causality [45]
Pu-erh/dark tea	Product- and enterotype-specific genus-level changes; altered BSH-related functions	Bile acids, theabrownin-derived products	FXR–FGF15/19 and cholesterol/lipid pathways	Strong mechanistic animal data; limited standardized human trials [24,46]

Abbreviations: BSH, bile salt hydrolase; FGF15/19, fibroblast growth factor 15/19; FXR, farnesoid X receptor; SCFA, short-chain fatty acid; TGR5, Takeda G-protein-coupled receptor 5. Taxonomic resolution is reported exactly as in the source studies; genus-level findings were not converted into species-level claims.

**Table 6:** Comparison of tea combined with dietary control, acute exercise, and chronic exercise training.

Combination	Primary Mechanism	Time Pattern	Evidence Strength	Effect-Size Interpretation	Clinical/Research Note
Tea + dietary energy control	Lower beverage energy; possible enzyme, microbiota and satiety support	Chronic; weeks to months	Moderate for structured polyphenol-rich dietary patterns; tea-specific contribution uncertain	Potentially meaningful when tea replaces caloric beverages; isolated effect small	DIRECT-PLUS supports the overall pattern, not tea alone [69,70]
Tea + acute exercise	Caffeine-driven stimulation and transient fat oxidation	Acute, pre-exercise	Low–moderate for acute physiology	Does not establish chronic weight loss	Consider caffeine tolerance, sleep and gastrointestinal effects [23,52]
Tea + chronic exercise training	Possible small additive effect on fat mass and lipids	Chronic; ≥4 weeks	Low–moderate; small heterogeneous RCTs	Meta-analysis indicates minimal additional weight effect	Exercise remains the primary intervention [68]
Tea + medical obesity treatment	Beverage substitution and dietary adherence; interactions possible	Chronic	Insufficient direct evidence	Unknown incremental effect	Review medications, liver risk, iron status and caffeine

## Data Availability

No new data were created or analyzed in this study. Data sharing is not applicable to this article.
